# Prevalence Rates of Pneumococcal Vaccination in IBD and 30-Day Clinical Outcomes in Patients With IBD and Pneumococcal Disease Stratified by Receipt of Pneumococcal Vaccination: A Multi-Network Study

**DOI:** 10.1093/crocol/otad048

**Published:** 2023-12-08

**Authors:** Andrew Ford, Vibhu Chittajallu, Jaime Abraham Perez, Scott Martin, Motasem Alkhayyat, Maneesh Dave, Edith Y Ho, Preetika Sinh, Vu Nguyen, Gregory Cooper, Jeffry Katz, Fabio Cominelli, Miguel Regueiro, Emad Mansoor

**Affiliations:** Department of Internal Medicine, Cleveland Clinic Foundation, Cleveland, OH, USA; Digestive Health Institute, Case Western Reserve University/University Hospitals Cleveland Medical Center, Cleveland, OH, USA; Clinical Research Center, University Hospitals Cleveland Medical Center, Cleveland, OH, USA; Clinical Research Center, University Hospitals Cleveland Medical Center, Cleveland, OH, USA; Department of Gastroenterology, Hepatology, and Nutrition, Digestive Disease and Surgery Institute, Cleveland Clinic Foundation, Cleveland, OH, USA; Division of Gastroenterology and Hepatology, Department of Internal Medicine, UC Davis Medical Center, UC Davis School of Medicine, Sacramento, CA, USA; Division of Gastroenterology and Hepatology, Department of Medicine, Stanford University School of Medicine, Stanford, CA, USA; Division of Gastroenterology and Hepatology, Medical College of Wisconsin, Milwaukee, WI, USA; Digestive Health Institute, Case Western Reserve University/University Hospitals Cleveland Medical Center, Cleveland, OH, USA; Digestive Health Institute, Case Western Reserve University/University Hospitals Cleveland Medical Center, Cleveland, OH, USA; Digestive Health Institute, Case Western Reserve University/University Hospitals Cleveland Medical Center, Cleveland, OH, USA; Digestive Health Institute, Case Western Reserve University/University Hospitals Cleveland Medical Center, Cleveland, OH, USA; Department of Gastroenterology, Hepatology, and Nutrition, Digestive Disease and Surgery Institute, Cleveland Clinic Foundation, Cleveland, OH, USA; Digestive Health Institute, Case Western Reserve University/University Hospitals Cleveland Medical Center, Cleveland, OH, USA

## Introduction

*Streptococcus pneumoniae* is a pathogen responsible for a number of illnesses including pneumonia, sepsis, and meningitis. Approximately 150 000 hospitalizations yearly in the United States occur from pneumococcal pneumonia, with a 5%–7% case fatality rate.^1^ Patients with inflammatory bowel disease (IBD), both Crohn’s disease (CD) and ulcerative colitis (UC), are at increased risk for developing pneumococcal disease due to their altered immune systems and treatment with immunosuppressive agents.^2,3^ This elevated risk has led to the recommendation that patients with IBD be vaccinated against streptococcal disease, particularly if on immunosuppression.^4^ Despite this recommendation, prior work has shown highly variable rates of vaccination among IBD patients ranging from 10% to 60%.^5^ Given the highly variable rates of pneumococcal vaccination in IBD reported in literature, we sought to characterize the rates of pneumococcal vaccination utilization in IBD patients utilizing a large population-based database. Additionally, we assessed clinical outcomes of rates of hospitalization, ICU admission, mechanical ventilation, and all-cause mortality within 30 days of pneumococcal infection among IBD patients with pneumococcal disease, stratified by vaccination.

## Methods

A retrospective cohort study was performed using TriNetX (Cambridge, MA), a multi-institutional database containing data from over 70 million patients across 58 US healthcare organizations between 2002 and 2022. Patients with IBD were defined as individuals carrying a diagnosis of UC or CD per ICD-10-CM coding and a prescription for at least 1 IBD-specific medication identified utilizing LOINC codes: Nonadvanced therapies: Mesalamine, sulfasalazine, balsalazide, budesonide; advanced therapies: azathioprine, methotrexate, 6-mercatopurine, infliximab, certolizumab, golimumab, adalimumab, vedolizumab, ustekinumab, tofacitinib, upadacitinib, and risankizumab. Pneumococcal vaccination was defined as recorded pneumococcal polysaccharide vaccine (PPSV23), pneumococcal conjugate vaccine (PCV13), or encounter for pneumococcal vaccine administration. We evaluated overall rates of vaccination among IBD patients and stratified them by 10-year age intervals, race, sex, and risk factors for severe pneumonia.

For outcomes, index event was pneumococcal infection occurrence defined by ICD coding for pneumonia (ICD-10-CM J13), sepsis (A41), or meningitis (G03) due to *Streptococcus pneumoniae*. Patients with IBD were then stratified by receipt of pneumococcal vaccine (case group: IBD patients with pneumococcal vaccination) or not (control group: IBD patients without pneumococcal vaccination). We then assessed outcomes among IBD patients with pneumococcal disease. Coprimary outcomes included rates of hospitalization, ICU admission, mechanical ventilation, and all-cause mortality within 30 days of pneumococcal infection.

Patients were then 1:1 propensity score matched by demographics including age, race, and sex, and risk factors for developing severe pneumonia including obesity, chronic obstructive pulmonary disease (COPD), asthma, and smoking. Odds ratios (ORs) were calculated for each outcome compared between the vaccinated and unvaccinated patients. For continuous data, analyses were conducted using independent *t*-tests, while chi-square tests were performed for categorical data. All tests were 2-tailed with an alpha level of .05.

## Results

**Figure 1.**
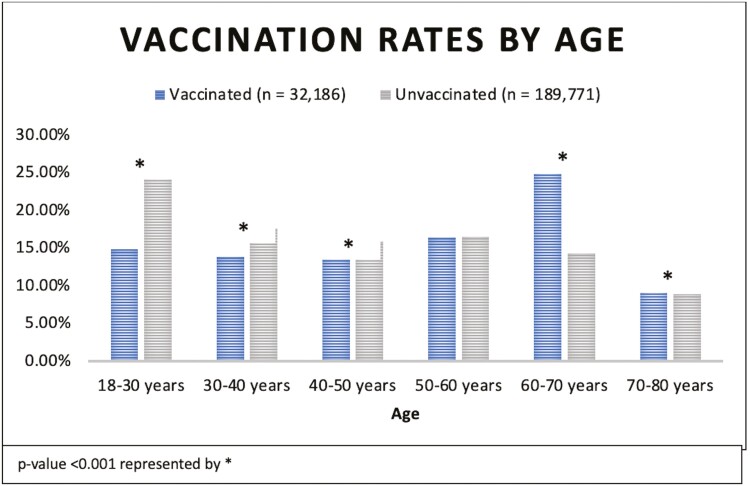
Pneumococcal vaccination rates in patients with inflammatory bowel disease stratified by age.

We identified 221 957 adult patients with IBD: 32 186 vaccinated patients (15%) and 189 771 unvaccinated patients (85%). Vaccination rates were lower in younger age groups 18–30 years old, 30–40, and 40–50 while they were higher in older age groups 60–70 and 70–80 (Figure 1). There were no significant differences in vaccination rates between groups based on sex or race (Caucasian vs African American). The presence of risk factors for severe pneumonia was associated with a higher likelihood of vaccination: smoking (15.0% vs 4.7%), COPD (9.2% vs 3.1%), asthma (16.4% vs 5.6%), alcohol dependence (4.3% vs 1.4%), and BMI > 25 (18.1% vs 5.6%; all *p*-values <.001) (Table 1).

**Table 1.** Pneumococcal vaccination rates in patient with IBD stratified by risk factors for severe pneumonia p value.

Variable	Vaccinated (*n* = 32 186)	Unvaccinated (*n* = 189 771)	*p* Value
Smoking	4827 (15.0%)	8767 (4.7%)	<.001
COPD	2942 (9.2%)	5783 (3.1%)	<.001
Asthma	5250 (16.4%)	10 462 (5.6%)	<.001
Alcohol dependence	1368 (4.3%)	2513 (1.4%)	<.001
BMI > 25	5816 (18.1%)	10 401 (5.6%)	<.001

Abbreviations: BMI, body mass index; COPD, chronic obstructive pulmonary disease; IBD, inflammatory bowel disease.

With respect to outcomes of pneumococcal disease in IBD patients, we found 28 638 patients with IBD and pneumococcal infection: 7742 vaccinated patients (27%) and 20 986 unvaccinated patients (73%) (Table 2). After propensity score matching, 7710 patients were included in each cohort. Vaccinated patients had lower odds of hospitalization (OR 0.71, 95% confidence interval [CI] 0.67–0.76), ICU admission (OR 0.85, 95% CI 0.77–0.93), mechanical ventilation (OR 0.84, 95% CI 0.73–0.97), and mortality (0.65, 95% CI 0.56–0.76) within 30 days of pneumococcal infection (Table 3).

**Table 2.** Demographic distribution (race, gender) and prevalence of risk factors for severe pneumococcal infection in index cohort of IBD patients with pneumococcal infection stratified by vaccination status, prior to propensity score matching p value.

Variable	Pneumococcal vaccination (*n* = 7719)	No pneumococcal vaccination (*n* = 20 647)	*p* Value
Age (years) ± SD	56.9 ± 16.8	55.2 ± 17.2	<.001
Female (%)	4398 (56.9%)	11 316 (54.8%)	.002
Caucasian	6369 (82.5%)	16 523 (80.0%)	<.001
African American	956 (12.4%)	2261 (11.0%)	.001
Asian	53 (0.7%)	196 (0.9%)	.035
Native American	17 (0.2%)	52 (0.3%)	.630
Obese	2065 (26.8%)	4104 (19.9%)	<.001
COPD	1847 (23.9%)	4161 (20.2%)	<.001
Asthma	2101 (27.2%)	4182 (20.3%)	<.001
Nicotine dependence	2073 (26.9%)	4804 (23.3%)	<.001

Abbreviations: COPD, chronic obstructive pulmonary disease; IBD, inflammatory bowel disease; SD, standard deviation.

**Table 3.** 30-Day outcomes after pneumococcal infection in IBD patients stratified by vaccination status, after propensity score matching for demographics and risk factors for severe pneumococcal infection p value.

Outcome	Pneumococcal vaccination (*n* = 7710)	No pneumococcal vaccination (*n* = 7710)	Odds ratio	95% CI
Hospitalization	2790	3413	0.71	0.67–0.76
ICU admission	1059	1221	0.85	0.77–0.93
Mechanical ventilation	372	437	0.84	0.74–0.97
Mortality	279	421	0.65	0.56–0.76

Abbreviations: CI, confidence interval; IBD, inflammatory bowel disease.

Of the 32 186 vaccinated patients, of those prescribed IBD medications, 12 199 (37.9%) had received nonadvanced therapies, while 19 987 (62.1%) had received advanced therapies. When stratified by age, younger patients were vaccinated more frequently if they had a history of advanced therapy prescription compared to patients not on advanced therapy (ie, among vaccinated patients 18–30 years old, 4337 [89.7%] had advanced therapy compared to 497 [10.3%] with nonadvanced; Table 4). This trend of higher proportion of younger patients with IBD being vaccinated if they were on advanced therapies continued from ages 18–30 to 51–60, after which therapy did not influence rates of pneumococcal vaccination in IBD.

**Table 4.** Medications prescribed among patients having received pneumococcal vaccination p value.

Age range	18-30 years	31–40 years	41–50 years	51–60 years	61–70 years	71–80 years	81–90 years
Nonadvanced therapy^*^ (%)	497 (10.3%)	759 (17.1%)	1088 (24.5%)	2019 (37.3%)	4621 (56.1%)	2553 (65.4%)	454 (71.8%)
Advanced therapy^†^ (%)	4337 (80.7%)	3686 (82.9%)	3348 (75.5%)	3398 (62.7%)	3612 (43.9%)	1349 (34.6%)	178 (28.2%)

^*^Nonadvanced therapies include mesalamine, sulfasalazine, balsalazide, and budesonide.

^†^Advanced therapies include azathioprine, methotrexate, 6-mercatopurine, infliximab, certolizumab, golimumab, adalimumab, vedolizumab, ustekinumab, tofacitinib, upadacitinib, and risankizumab.

## Discussion

This is one of the largest studies to date evaluating the rates of pneumococcal vaccination uptake in IBD and its efficacy. Our finding of a 15% vaccination rate is on the lower end of rates of 10%–60%^5^ as reported in prior studies on pneumococcal vaccination among IBD patients; Case et al. found a vaccination rate of 20% among IBD patients, while Love et al. found a rate of 42%.^6,7^ This difference may stem from the former studies’ use of Veterans’ Affairs (VA) data, while ours incorporates data from across a wider array of healthcare systems with potentially varying vaccination practices. However, our observation that vaccination was higher among older patients is in line with this previous work.^7^ Younger patients who did receive vaccination were more likely to have a history of receiving advanced therapies, which may reflect awareness of the immunosuppression that many of these agents carry and is in line with current ACIP recommendations.^8–13^ Most importantly, given the significantly large sample size of 221 957 patients in this study, our findings have greater validity compared to prior studies.

As for outcomes of pneumococcal disease among IBD patients, we found that pneumococcal vaccination was significantly protective against hospitalization, ICU admission, mechanical ventilation, and mortality within 30 days of pneumococcal disease diagnosis. This finding is similar to Love et al.’s recent work demonstrating that vaccination was protective against severe pneumococcal disease among VA patients.^6^

This is the largest study to date assessing rates of pneumococcal vaccination and outcomes of pneumococcal disease among IBD patients to our knowledge. Aside from a large sample size, additional strengths of our study include propensity score matching to control for potential confounding variables. Limitations of our study include the use of billing codes, retrospective cohort analysis, and the inability to detect vaccination status if patients received vaccination outside of HCOs covered by TrinetX which may have led us to underestimate vaccination rates. The former-most limitation is mitigated by including IBD-specific medication prescription as a factor in defining IBD, thereby increasing the sensitivity. However, this strategy limits the ability to determine the effect of IBD-specific medications on the development of severe pneumococcal disease. Further, previous work has demonstrated that administrative data such as billing codes can be highly accurate in identifying patients with pneumococcal disease, particularly when combined with prescription codes.^14^

In conclusion, in the largest retrospective cohort study to date on pneumococcal vaccination rates in IBD, we found an overall vaccination rate of 15% and significantly lower rates of vaccination against pneumococcal disease among younger patients, though younger patients who were vaccinated tended to have a history of advanced IBD therapy. It additionally showed an increased risk of hospitalization, ICU admission, mechanical ventilation, and mortality among IBD patients not vaccinated against pneumococcal disease. These findings call for the implementation of system-based programs to improve rates of pneumococcal vaccination in IBD to prevent adverse outcomes.

## Data Availability

Data not publicly available, though can be requested from TriNetX (live.trinetx.com). This may be at additional cost, data-use agreement may be required, and identifiable information cannot be obtained.
